# A Patient-Centered PaTH to Address Diabetes: Protocol for a Study on the Impact of Obesity Counseling

**DOI:** 10.2196/12054

**Published:** 2019-04-04

**Authors:** Jennifer L Kraschnewski, Lan Kong, Erica Francis, Hsin-Chieh Yeh, Cindy Bryce, Jennifer Poger, Erik Lehman

**Affiliations:** 1 Penn State Health College of Medicine Penn State University Hershey, PA United States; 2 Johns Hopkins School of Medicine Johns Hopkins University Baltimore, MD United States; 3 University of Pittsburgh Department of Public Health Pittsburgh, PA United States

**Keywords:** diabetes complications, obesity, electronic medical record

## Abstract

**Background:**

Overweight and obesity are America’s number one health concern. The prevalence of obesity in the United States is greater than 36%, a rate that has doubled since 1970. As the second most preventable cause of death, obesity is a risk factor for diabetes, cardiovascular disease, stroke, and cancer, all major causes of death. Primary care clinics may be an ideal setting for weight control interventions to help manage and prevent diabetes. For this reason, the Centers for Medicare and Medicaid Services (CMS) implemented a health care procedure coding system code for intensive behavioral therapy (IBT) for obesity within primary care in 2012 to facilitate payment for addressing obesity, which was followed by broader coverage by most insurers for IBT for adults in 2013. However, the impact of this coverage on patient-centered outcomes is largely unknown.

**Objective:**

The overarching goal of this study is to understand the comparative effectiveness of obesity counseling as covered by CMS and other insurers in improving weight loss for adults either with or at increased risk for type 2 diabetes.

**Methods:**

This study leverages the novel infrastructure of the Patient-Centered Outcomes Research Institute–funded PaTH Clinical Data Research Network. The PaTH network is comprised of Geisinger Health System, Johns Hopkins University, Johns Hopkins Health System, Lewis Katz School of Medicine at Temple University, Temple Health System, Penn State College of Medicine, Penn State Milton S Hershey Medical Center, University of Pittsburgh, UPMC and UPMC Health Plan, and the University of Utah. Electronic health record (EHR) data will originate from the 6 PaTH health systems. Specifically, we will (1) evaluate the impact of broader preventive service coverage for obesity screening and counseling on weight loss, diabetes incidence, and diabetes outcomes in patients with diabetes or at increased risk for diabetes (defined by body mass index [BMI] ≥25). We will determine how the annual probability of receiving obesity and/or nutritional counseling changed pre- and postpolicy across all insurers in a cohort of patients with diabetes and at high risk for diabetes. We will (2) compare patient weight loss and diabetes-related outcomes among those who receive obesity screening and counseling with those who do not, following implementation of preventive service coverage. We will examine postpolicy impact of obesity screening and counseling in a cohort of patients with diabetes and at increased risk for diabetes. Specific outcomes to be examined include weight loss, diabetes incidence, and diabetes outcomes. Exploratory outcomes will include patient-reported outcomes. Furthermore, we will determine patient characteristics, including demographics, and practice characteristics, including provider type.

**Results:**

Our PCORI-funded study is underway. To date, we have obtained our second data extraction from the PaTH CDRN and are performing data editing and cleaning. Next steps include analysis of early policy change.

**Conclusions:**

Given patients who are overweight are at highest risk for diabetes, improved weight management services could prevent diabetes and its negative health outcomes. Comparing weight and diabetes outcomes in 3 states using EHRs and claims data before and after this policy was implemented using the PaTH Network will allow important insight into policy effectiveness.

**International Registered Report Identifier (IRRID):**

DERR1-10.2196/12054

## Introduction

### Background

Overweight and obesity are America’s number one health concern. The prevalence of obesity in the United States is greater than 36% [1], which is far above the Healthy People 2020 objective of less than 30.5% [2]. Perhaps, the most concerning is the rate in which obesity has increased, having doubled since 1970 [3]. As the second most preventable cause of death [4], obesity is a risk factor for diabetes, cardiovascular disease, stroke, and cancer, all major causes of death in the United States [5]. Addressing obesity through lifestyle interventions decreases the risk of developing type 2 diabetes, a disease which affects over 29 million people (9.3% of the US population) [6]. Diabetes is associated with serious complications, including cardiovascular disease, blindness, renal failure, and lower extremity amputation. Although complications are preventable with proper medical and lifestyle management, including weight loss, nearly half of the patients with diabetes do not maintain adequate glycemic control [7].

Primary care clinics may be an ideal setting for weight control interventions. More than 80% of Americans see a primary care physician (PCP) regularly, and access to primary care is expected to increase with health care reform [8]. Furthermore, as PCPs identify and treat a multitude of conditions affected by being overweight, including diabetes, they are ideally positioned to best engage their patients in weight management. In 2012, the Centers for Medicare and Medicaid Services (CMS) implemented a health care procedure coding system code for intensive behavioral therapy (IBT) for obesity within primary care settings to facilitate payment for addressing obesity, which was followed by universal coverage among nongrandfathered private plans without cost sharing for adults of all ages in 2013, a key provision of the Affordable Care Act [9-11]. The rate of uptake of the Medicare obesity benefit within the first 2 years of implementation was small (0.10% and 0.17%, respectively) among beneficiaries. However, the updated impact of this policy coverage on patient-centered outcomes across insurers remains largely unknown.

This study leverages the novel infrastructure of the Patient-Centered Outcomes Research Institute (PCORI)–funded PaTH Clinical Data Research Network (CDRN), a partnership of 4 mid-Atlantic academic health systems (Penn State Hershey Medical Center, University of Pittsburgh Medical Center, Temple Health System, and Johns Hopkins Health System) that has established governance to operate as an integrated research network. In 2015, the University of Utah and Geisinger Health System also joined PaTH, creating an electronic health record (EHR)–based data infrastructure across 3 states (Maryland, Pennsylvania, and Utah).

This study is significant for several reasons. First, diabetes is a leading public health concern and is associated with significant economic burden. Recent health policy changes (eg, CMS coverage) are expected to impact diabetes outcomes, and this study will capture differences in these outcomes through varied state implementation. Understanding effects on diabetes outcomes can inform future policies to improve overall diabetes care for patients. Second, this study focuses on the influence of policy-level factors for diabetes management. Poor outcomes are preventable but require complex medical and lifestyle management, including careful diet modification, medication use including oral pills and/or insulin injections, blood glucose self-monitoring, frequent medical visits and laboratory testing, cholesterol management, weight management, and physical activity. The ability of individuals with diabetes to effectively manage their diabetes is multifactorial, influenced by individual-, social-, and policy-level factors [12]. Finally, we have an additional focus on rural/urban differences in provision of obesity screening and counseling and the resultant impact on weight loss and diabetes incidence.

### Objectives

The overarching goal of this study was to understand the comparative effectiveness of obesity counseling as covered by CMS in improving weight loss for adults either with or at increased risk for type 2 diabetes. CMS and most insurers now include obesity screening and counseling benefits, with no cost sharing to patients [9]. As patients who are overweight are at highest risk for diabetes, improved weight management services could prevent diabetes and its negative health outcomes. CMS beneficiaries with obesity are eligible for up to 20 face-to-face visits for weight counseling in the primary care setting, although total visits may vary for other insurers. We will compare weight and diabetes outcomes in 3 states using EHRs and claims data before and after this policy was implemented. Using the PaTH CDRN infrastructure, the study will aim to do the following:

- Aim 1: The study will evaluate the impact of broader preventive service coverage for obesity screening and counseling on weight loss, diabetes incidence, and diabetes outcomes in patients with diabetes or at increased risk for diabetes (defined by body mass index [BMI] ≥25). We will determine how the annual probability of receiving obesity counseling (as defined by Common Procedural Treatment [CPT] codes G0447, G0473, S9470, and/or S9449) changed pre- and postpolicy across all insurers in a cohort of patients with diabetes and at increased risk for diabetes. We hypothesize that individual patients are more likely to receive counseling following coverage implementation. Furthermore, we hypothesize that patients who receive a greater number of face-to-face visits will have greater weight loss compared with those who receive fewer visits. Exploratory outcomes will include patient-reported outcomes (PROs).
- Aim 2: The study will compare patient weight loss and diabetes-related outcomes among those who receive obesity screening and counseling with those who do not, following implementation of preventive service coverage. We will examine postpolicy impact of obesity screening and counseling in a cohort of patients with diabetes and at increased risk for diabetes. Specific outcomes to be examined include weight loss, diabetes incidence, and diabetes outcomes (including hemoglobin A_1c_ [HbA_1c_], controlled blood pressure, and use of a statin medication). Exploratory outcomes will include PROs. Furthermore, we will determine patient characteristics, including demographics (age, race/ethnicity, and rurality), and practice characteristics, including provider type, and their impact on receiving/providing obesity screening and counseling. Understanding patient and practice characteristics most likely to engage in obesity counseling can identify best practices and inform how to increase engagement by both patients and providers.

## Methods

### Preliminary Studies

#### PaTH Clinical Data Research Network

The PaTH CDRN provides an infrastructure for pragmatic clinical trials and observational studies that require populations beyond a single health system to answer important patient-centered clinical and health services questions [13]. Funded by the PCORI in March 2014, the PaTH CDRN is one of 11 CDRNs across the country. Along with 18 Patient-Powered Research Networks, these 11 CDRNs form the National Patient-Centered Clinical Research Network (PCORnet)—a national network for conducting clinical outcomes research [14]. The goal of PCORnet is to improve the nation’s capacity to conduct comparative effectiveness research by creating a large, highly representative network from which to draw data, while protecting patient privacy and ensuring data security.

The patients in the PaTH network are diverse—22% are aged 17 years or younger and 20% are aged 65 years or older. Over 25% are nonwhite and 20% have public insurance (excluding Medicare) or no insurance. The organizations are also diverse and are affiliated with community-based hospitals and outpatient practices in addition to their academic hospitals. Other facilities include rehabilitation hospitals, dialysis centers, fitness and wellness centers, psychiatric hospitals, ambulatory surgery centers, and home health care support.

PaTH leverages health-related data from (1) EHRs, (2) PROs, (3) insurance claims data, and (4) biospecimen data. The PaTH data that will be used in the study will be limited to EHR data and claims data.

The PaTH network has also established a centralized process for institutional review board (IRB) reviews. Creating separate IRB protocols with different formats and procedures to be reviewed by separate IRBs would be an inefficient and ineffective process. This problem has been recognized by the National Institutes of Health, which promotes use of a single IRB in multisite clinical research studies to reduce duplication of effort, speed-up the initiation of important research, and save time and resources [15]. To this end, the PaTH network has established a reliance agreement naming Johns Hopkins’ IRB as our central IRB of record. Under the reliance agreement, the other institutions agree to allow the Johns Hopkins’ IRB to review the study protocol and to honor the approval of the protocol. To ensure that each PaTH institution would have input into the review process, we convened the PaTH Network Protocol Review Committee (PNPRC). A total of 2 IRB members from each institution serve on the PNPRC, an IRB member and a community member, currently totaling 8 members. After the PNPRC approves a PaTH protocol, it is then submitted to the Johns Hopkins’ IRB for centralized review.

### Data Sources for All Aims

#### Electronic Health Records/PaTH Clinical Data Research Network

EHR data will originate from the 6 PaTH health systems. These health systems have greater than 13 million patients with at least one encounter and 5 million active patients in their EHR systems (see Table 1).

PaTH has united previously disconnected health care systems with a common, scalable data architecture. Our health systems employ the 2 most commonly used EHR systems nationwide—Epic and Cerner. Penn State uses Cerner; University of Pittsburgh Medical Center (UPMC) uses Epic for its outpatient EHRs and Cerner for its inpatient EHRs; and Temple, Johns Hopkins, Geisinger, and the University of Utah use Epic. The health systems also incorporate data from ancillary Information Technology (IT) systems including Eclipsys, General Electric, AllScripts, and Phillips. Each EHR system has undergone extensive customization during their lifetimes, creating disparate systems with inherent interoperability gaps across all areas including diagnosis, lab results, and patient demographic data.

All health systems are using, or will use, the following standards to achieve semantic interoperability for their EHRs and ancillary systems: LOINC for encoding laboratory tests; Systematized Nomenclature of Medicine (SNOMED) for medical terminologies; CPT and International Classification of Diseases (ICD)-9 and ICD-10 for encoding problems, diagnoses, and procedures; RxNORM for encoding medications; and DICOM for transmitting radiologic images. Given the heterogeneity of the EHRs, the PaTH network sought an existing solution that permits intersystem syntactic interoperability and leverages previous investments and expertise.

**Table 1:** Patient population overview at individual clinical sites.

Clinical Site Criteria^a,b^	Penn State Hershey	Pitt/UPMC^c^	TUHS^d^	Hopkins	University of Utah
EHR^e^ platform	Cerner	Cerner (inpatient); Epic (outpatient)	Epic	Epic	Epic
Distinct patients with at least 1 encounter or record in HER, n	615,012	5,537,583	457,388	4,800,000	1,602,245
Active patients with data in EHR, n	520,310	1,880,457	323,682	1,764,221	581,568

^a^Geisinger Health System was added to the PaTH Clinical Data Research Network (CDRN) after submission of this proposal.

^b^Data pulled in 2015 from i2b2 at each site.

^c^UPMC: University of Pittsburgh Medical Center.

^d^TUHS: *Temple University Health System.*

^e^EHR: electronic health record.

Each health system’s efforts to utilize standard vocabularies and formats can significantly narrow the gap of interoperability, but the final bridge is the PCORnet-specified Common Data Model (CDM) [16]. The PCORnet CDM enables us to transform each health care system’s dialect into a common language standardized on the meaningful use–recommended vocabularies (SNOMED, RxNORM, and LOINC). The PCORI CDM provides specifications for what common data elements each CDRN must include at a minimum and standardizations for how they are to be named and mapped in a consistent format (eg, with the same variable name, precision, and other metadata) within standard health care terminologies to ensure interoperability within sites and between networks. The PaTH network is in compliance with PCORnet CDM version 1.0 and is moving to conform to the recently released CDM version 2.0. All of the 11 PCORnet CDRNs are required to define at least 1 million patients by Fall 2015, which includes patients with complete data and data specifications in compliance with the latest update of the PCORnet CDM creating a large network of networks, all with data mapped to a consistent format.

Once the PaTH research team cooperatively identifies data elements appropriate for the prespecified research questions, extract, transform, load teams at each site extract these data elements from their EHRs or ancillary systems, deidentify the data, map these data to standard vocabularies as specified by the PCORnet CDM and any additional PaTH data element specifications, and load the data to Pittsburgh’s Comparative Effectiveness Research Core Data Center (CERC-DC), which provides secure data storage and high-throughput computing.

**Figure 1:**
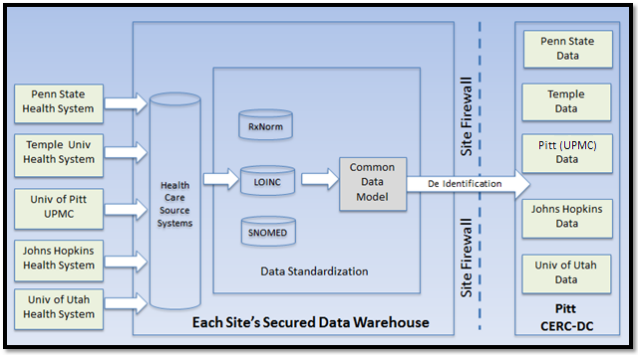
PaTH network data integration scheme for the proposed project. CERC-DC: Comparative Effectiveness Research Core Data Center; SNOMED: Systematized Nomenclature of Medicine; UPMC: University of Pittsburgh Medical Center.

The informatics design of PaTH has 2 main features: (1) the ability to support researchers to easily perform exploratory research queries (ie, counts) through the distributed data network and (2) the ability to support use of aggregated deidentified data that conform to our shared PaTH information model. Currently, the PaTH network uses the University of Pittsburgh CERC-DC to house these aggregated datasets. One important feature in this model is that it incorporates authorization and audit mechanisms to ensure that each site retains adequate control and logs of their data. The additional data integration with contextual data and the associated data flow for this study is described in Figure 1.

#### Claims Data

A limitation of EHR data is the uncertainty of its completeness, that is, when a patient receives medical care outside of the health system or is hospitalized while away on vacation. However, claims data can capture clinical encounters that occur outside of our 6 health systems. Claims data also provide other supplementary information—for example, the EHR only tells us a patient was prescribed a statin medication but not whether the patient picked it up from the pharmacy. The insurance data can verify whether a pharmacy claim for the medication was processed, indicating the patient received the medication.

#### Secure Sharing of Deidentified Integrated Patient Data for Analysis

The PaTH network has established an operational data infrastructure with the necessary technical safeguards as agreed upon in the PaTH Data Use Agreement for sharing and analyzing data while addressing data confidentiality and security concerns. PaTH has deployed 2 mechanisms for storing, protecting, and sharing data (as described previously under PaTH governance and regulatory issues): (1) data with protected health information (PHI) are stored and protected behind each institution’s firewall in the distributed data network and (2) deidentified data are sent to the PaTH data center at the University of Pittsburgh (CERC-DC). Once data integration is accomplished at each site, sites will remove all PHI and send the deidentified version of the integrated data to the CERC-DC through PaTH’s virtual private network/secure file transfer protocol, which has been operational for data transmission since November 2014. Data analysis will then be performed via secure remote computing using standard statistical software packages (eg, SAS developed by the SAS Institute).

#### PaTH Patients With Diabetes and At Increased Risk for Diabetes

The PaTH CDRN data infrastructure is designed to support a broad range of research topics, with the ability to define specific patient cohorts when needed to support specific use case research questions. This study utilizes 2 patient cohorts: (1) diabetes cohort and (2) at increased risk for diabetes cohort. As demonstrated in Tables 2 and 3, the PaTH network includes over 328,000 patients with a diagnosis of diabetes (defined as aged 18 years and older with a diagnosis of diabetes mellitus—ICD-9 250.xx) and over 2 million patients at increased risk for diabetes (defined as aged 18 years and older with a BMI of ≥25). We recognize use of BMI ≥25 is a limited definition for patients at increased risk for diabetes, particularly when used across all racial and ethnic groups. However, given that only patients with obesity (BMI ≥30) would be eligible for IBT, this threshold allows for appropriate inclusion of patients with future opportunity for narrowing the definition. In addition, we have demonstrated our preliminary results of several of the Healthy People 2020 objectives, which will serve as outcomes for the study (see Table 4), indicating the feasibility and accessibility of these data. For this study, receipt of IBT will include the presence of the G0447, G0473, S9470, and/or S9449 CPT codes with a diagnosis of obesity (278.00, 278.01, 278.03, and 278.01, respectively; V85.3-V85.4) consistent with regulatory requirements.

**Table 2:** Preliminary data of PaTH patients with diabetes.

Patient characteristics^a,b^		Penn State University (N=25,219), n (%)	University of Pittsburgh (N=150,589), n (%)	Johns Hopkins University (N=60,324), n (%)	Temple University (N=40,536), n (%)	University of Utah (N=51,787), n (%)	PaTH total (N=328,455), n (%)
**Insurance type**							
	Private	9874 (38)	60,223 (40)	32,371 (54)	5261 (13)	21,264 (41)	128,993 (39)
	Medicaid	1890 (8)	10,165 (7)	727 (1)	21,759 (54)	4189 (8)	38,730 (12)
	Medicare	12,998 (52)	54,675 (36)	22,826 (38)	10,916 (27)	23,997 (46)	125,412 (38)
	Uninsured	457 (2)	11,625 (8)	485 (1)	2599 (6)	2337 (5)	17,503 (5)
**Race**							
	White	21,780 (86)	116,056 (77)	32,544 (54)	14,347 (35)	39,580 (76)	224,307 (68)
	African American	1679 (7)	15,336 (10)	20,053 (33)	14,656 (36)	944 (2)	52,668 (16)
	Other	1760 (7)	19,197 (13)	7727 (13)	11,533 (29)	11,263 (22)	51,480 (16)
	Hispanic ethnicity	1054 (4%)	697 (0.5)	2002 (3)	7356(18)	6077 (12)	17,186 (5)
Female gender		12,014 (48)	70,623 (47)	30,912 (51)	22,485 (55)	25,727 (50)	161,761 (49)

^a^Geisinger Health System was added to the PaTH Clinical Data Research Network (CDRN) after submission of this proposal.

^b^Data pulled in 2015 from i2b2 at each site.

**Table 3:** Preliminary data of PaTH patients at increased risk for diabetes.

BMI^a,b,^^c^	Penn State University (N=167,799), n (%)	University of Pittsburgh (N=950,020), n (%)	Johns Hopkins University (N=471,860), n (%)	Temple University (N=212,314), n (%)	University of Utah (N=260,506), n (%)	PaTH total (N=2,062,499), n (%)
25-29.9	69,353 (30)	433,799 (31)	226,113 (32)	92,807 (31)	122,583 (31)	944,655 (32)
30-34.9	48,353 (21)	268,236 (19)	128,799 (18)	60,330 (20)	87,023 (22)	592,741 (20)
35-39.9	25,388 (11)	128,113 (9)	58,072 (8)	29,930 (10)	70,774 (18)	312,277 (10)
40+	24,705 (11)	94,550 (7)	43,377 (6)	23,321 (8)	25,410 (7)	211,363 (7)

^a^Geisinger Health System was added to the PaTH Clinical Data Research Network (CDRN) after submission of this proposal.

^b^Data pulled in 2015 from i2b2 at each site.

^c^BMI: body mass index.

**Table 4:** Preliminary data of Healthy People 2020 objectives for PaTH patients with diabetes (N=328,455) and at increased risk for diabetes (N=2,062,499).

Patient characteristics^a,b^				Across PaTH, n (%)	Healthy People 2020		
					Baseline (%)	Goal (%)
**Patients with diabetes**						
	With controlled diabetes (ie, HbA_1c_^c^<7)			80,486 (25)	53.5	58.9
	With uncontrolled diabetes (ie, HbA_1c_>9)			47,701 (15)	17.9	16.1
	With controlled blood pressure (ie, <140/90)			123,038 (38)	51.8	57
	On a statin medication			132,841 (40)	—^e^	—^e^
	With annual urinary microalbumin			65,180 (20)	33.6	37
	With ≥2 HbA_1c_ values during past year			67,797 (21)	64.6	71.1
**Patients at increased risk for diabetes**						
	**BMI^d^**					
		18.5-24.9		934,664 (31)	30.8	33.9
		25-29.9		944,655 (32)	—	—
		30+ (obese)		1,116,381 (37)	33.9	30.5
	Percentage obese (BMI ≥30) who receive nutrition counseling (CPT^f^ codes 97802, 97803, 97804, G0270, G0271)			10,717 (1)	—	—
	Percentage without diagnosis of diabetes (ICD-9^g^ 250.xx) but with ≥1 A_1c_			179,412 (16)	—	—
	Percentage with diagnosis of hypertension (ICD-9 401) who receive nutrition counseling (CPT codes 97802, 97803, 97804, G0270, G0271)			6747 (<1)	—	—

^a^Does not include data from Geisinger.

^b^Data pulled in 2015 from i2b2 at each site.

^c^HbA_1c_: hemoglobin A_1c_.

^d^BMI: body mass index.

^e^Not applicable.

^f^CPT: Common Procedural Treatment.

^g^ICD-9: International Classification of Diseases, Ninth revision.

### Research Design

The overarching goal of this research was to understand the comparative effectiveness of obesity counseling as covered by CMS in improving weight loss for adults either with or at increased risk for type 2 diabetes**.** Using the PaTH Network infrastructure, we will examine the impact of the policies on a population of more than 328,000 patients with diabetes, as well as an additional 2,000,000 patients at increased risk for the development of diabetes.

#### Aim 1: Overview

Evaluate the impact of broader preventive service coverage for obesity screening and counseling on weight loss, diabetes incidence, and diabetes outcomes in patients with diabetes or at increased risk for diabetes (defined by BMI ≥25). We will determine how the annual probability of receiving obesity and/or nutritional counseling (as defined by CPT code) changed pre- and postpolicy across all insurers in a cohort of patients with diabetes and at increased risk for diabetes. We hypothesize that individual patients are more likely to receive counseling following coverage implementation. Furthermore, we hypothesize that patients who receive a greater number of face-to-face visits will have greater weight loss compared with those who receive fewer visits. Exploratory outcomes will include PROs, as outlined in Table 5.

Weight loss is an important patient-centered outcome, as nearly every patient with overweight/obesity desires weight loss and assistance from their physician but few currently receive it [17-21]. Furthermore, our CDRN patient partners indicated that weight loss and diabetes incidence are significant patient-centered outcomes.

HbA_1c_ also remains an important patient-centered outcome, given it is well-established that improved glycemic control results in prevention of serious complications (cardiovascular disease, blindness, renal failure, and lower extremity amputation) and is appropriate for the timeframe of the study. In addition, we will examine blood pressure control, use of a statin medication, and appropriate diabetic screening, given the importance of these guideline-recommended measures in diabetes care. Exploratory outcomes will include PROs (including *Patient-Reported Outcomes Measurement Information System*, Short Form-12, and Patient Heath Questionnaire) listed in Table 5, which are available at some of our sites across the CDRN.

**Table 5:** Outcomes for the diabetes and at increased risk for diabetes cohorts.

Outcomes		Definition	Notes
**Diabetes cohort**			
	Weight loss during counseling	Weight lost from first intensive behavioral therapy (IBT) visit to final IBT visit	Available at all PaTH sites
	Weight loss maintenance	Percentage of weight lost during program and maintained over remaining time period, reported by year	Available at all PaTH sites
	Patient-reported outcomes (PROs)	Short Form-12 (SF-12); Patient Health Questionnaire (PHQ-2, PHQ-8, PHQ-9; physical function; Sleep; Fruit and vegetable consumption; Social support; Physical activity; *Patient-Reported Outcomes Measurement Information System* *(* PROMIS; PROMIS 29, physical function, depression); Healthy lifestyles; Patient-reported medication reconciliation	Available at some sites—formal inventory of PROs will be collected at each institution at the beginning of the project, to be included as secondary outcomes
	Uncontrolled diabetes	Average A_1c_>9 or no A_1c_	Available at all PaTH sites
	Controlled blood pressure	Systolic blood pressure <140, diastolic blood pressure <90, averaged across values over a year	Available at all PaTH sites
	On a statin medication	Evidence of a statin medication on current electronic health record medication list	Available at all PaTH sites
	Receiving annual eye exam	Documentation of eye exam once in past year	Available at all PaTH sites
	Receiving annual urinary microalbumin test	Documentation of lab testing for urinary microalbumin at least once in past year	Available at all PaTH sites
	Lower extremity amputations	Documentation of procedure for lower extremity amputation or billing code through health plans in past year	Available at all PaTH sites
	Diabetes Service Use	-Clinic visit with primary or secondary diagnosis of diabetes; Emergency department visit with primary or secondary diagnosis of diabetes; Hospitalization with primary or secondary diagnosis of diabetes	Available at all PaTH sites
**At increased risk for diabetes cohort**			
	Weight loss during counseling	Weight lost from first IBT visit to final IBT visit	Available at all PaTH sites
	Weight loss maintenance	Percentage of weight lost during program and maintained over remaining time period, reported by year	Available at all PaTH sites
	Diabetes incidence	Percentage of patients who develop diabetes per year following weight counseling	Available at all PaTH sites
	PROs	-SF-12; PHQ-2, PHQ-8, PHQ-9; Physical function; Sleep; Fruit and vegetable consumption; Social support; Physical activity; PROMIS (PROMIS 29, physical function, depression); Healthy lifestyles; Patient-reported medication reconciliation	Available at some sites—formal inventory of PROs will be collected at each institution at the beginning of the project, to be included as secondary outcomes
**Exposure variables**			
	Individual level	-Sociodemographics (eg, age, sex, race, insurance status, and rural vs urban); Medical comorbidities	Available at all PaTH sites
	Provider/practice level	-Practitioner type (advanced practice vs MD/DO); Practitioner specialty; Practice size (number of providers); Practice type (multispecialty, academic); Practice setting (rural vs urban)	To be determined

##### Diabetes Cohort Definition

**Figure 2:**
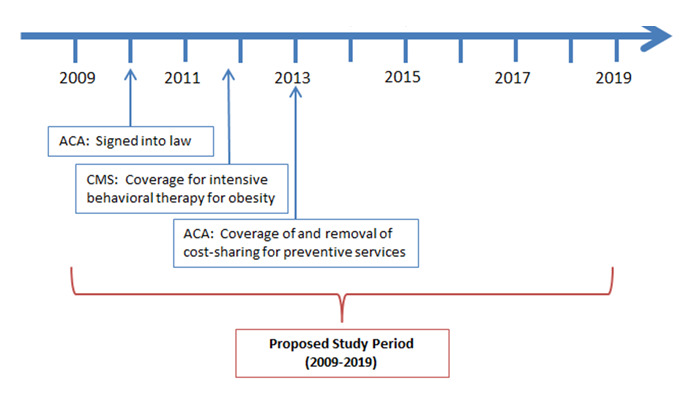
Timeline for Affordable Care Act and Centers for Medicare and Medicaid Services policy changes. ACA: Affordable Care Act; CMS: Centers for Medicare and Medicaid Services.

During year 1 of the proposed project, the investigative team, in collaboration with the PaTH Network, will identify a valid cohort of patients with type 2 diabetes. The cohort of patients under study will be defined as all patients aged 18 years and older with an indication of type 2 diabetes during the proposed study timeframe. Patients will be classified as having diabetes using a clinically validated algorithm: type 2 diabetes mellitus on the problem list, diabetes-specific medications, HbA_1c_ results >7.0%, or 1 inpatient diagnosis code or 2 out-patient diagnosis codes for type 2 diabetes (ICD-9 codes 250.xx). This algorithm has been shown to have 98% sensitivity and 98% specificity for diabetes when compared with the gold standard of manual chart review by a trained research nurse [22]. The diabetes cohort will be further limited to patients who will likely be captured in the PaTH EHRs or claims data so that outcome assessments can occur. Thus, we will further limit the diabetes cohort to patients who have either (1) had at least 2 outpatient primary care visits in 1 of the PaTH health systems in the past 3 years (since January 1, 2012) or (2) for whom claims data are available. The cohort will be dynamic, with new patients added into the cohort after 2015 as they meet the diabetes cohort definition prospectively. The observational period for the outcome variables will be for the 10-year period from 2009 to 2019, thus including 3 years of data before the first policy change (CMS instituting coverage for IBT for obesity) and 3 years after the last policy change (Pennsylvania Medicaid expansion) under study (Figure 2).

##### Definition and Measurement of Key Diabetes Outcomes and Covariates

Key individual-level diabetes outcomes relate to the Healthy People 2020 diabetes objectives (Table 6). Diabetes outcomes will be assessed through PaTH EHRs and supplemented by claims data when available. Key exposure variables will include individual-level variables (sociodemographics and medical comorbidities) and state of residence (to capture state-specific variation in policy implementation).

Following the definition of the diabetes cohort and key diabetes outcomes and covariates as described above, an initial extraction of variables will be conducted in year 2 of the proposed project for years 2009 to 2015. This early data extraction from the PaTH Network will allow for cohort validation and data cleaning and editing, as well as required programming and determination of the analysis models. We will utilize this initial data extraction in years 2 and 3 to analyze the impact of broader coverage for intensive behavioral counseling. The final data extraction will occur during the final quarter of year 4 of the proposed project, allowing for completion of a 10-year time period (2019).

As older adults have various degrees of comorbidity conditions, the American Diabetes Association (ADA) developed a framework (Table 7) considering treatment goals for glycemic control, blood pressure, and dyslipidemia in older adults with diabetes [23,24]. Therefore, we will conduct subgroup analysis in older adults according to these recommendations. In this population, individualized A_1c_ targets were recommended by ADA: <7.5%, 8%, and 8.5% for healthy, complex/intermediate, and very complex/poor health patients, respectively. However, the classification of health status was subjective, and not every patient will clearly fall into a specific category (eg, cognitive function and functional limitations). For the purpose of this study, we will conduct subgroup analysis in older adults using individualized A_1c_ targets based on presence of complications: individuals without diabetic-related complications (A_1c_ level, <7.5%) and those with diabetic-related complications (A_1c_ level, <8.0%). Complications may include arthritis, cancer, congestive heart failure, depression, emphysema, falls, hypertension, incontinence, stage 3 or worse chronic kidney disease, myocardial infarction, stroke, oxygen-dependent lung disease, chronic kidney disease requiring dialysis, or uncontrolled metastatic cancer. Those conditions may cause significant symptoms or impairment of functional status and significantly reduce life expectancy.

**Table 6:** Aim 1: key diabetes outcomes and covariates.

Diabetes outcomes (extracted annually)		Definition
**Individual-level outcomes**		
	Controlled diabetes (HP obj D-5)	Average A_1c_<7 (LOINC: 4548-4)
	Uncontrolled diabetes (HP obj D-5)	Average A_1c_>9 or no A_1c_ measurement in past year (LOINC: 4548-4)
	Controlled blood pressure (HP obj D-7)	SBP^a^<140, DBP^b^<90, averaged across values over year
	On a statin medication (HP obj D-6)	Evidence of a statin medication on current EHR^c^ medication list
	Receiving annual eye exam (HP obj D-10)	Documentation of eye exam once in past year
	≥2 A_1c_ tests each year (HP obj D-11)	Documentation of lab testing for A_1c_ (LOINC: 4548-4)
	Receiving annual urinary microalbumin test (HP obj D-12)	Documentation of lab testing for urinary microalbumin at least once in past year (LOINC: 14957-5)
	Lower extremity amputations (HP obj D-4)	Documentation of procedure for lower extremity amputation or billing code through health plans in past year
**Exposure variables**		
	Individual level	Sociodemographics (eg, age, sex, race, and insurance status); Medical comorbidities
	Policy level	CMS^d^ and other insurer implementation

^a^SBP: systolic blood pressure.

^b^DBP: diastolic blood pressure.

^c^EHR: electronic health record.

^d^CMS: Centers for Medicare and Medicaid Services.

**Table 7:** American Diabetes Association Framework for considering treatment goals in older adults with diabetes.

Patient characteristics	ADA^a^ rationale	Reasonable A_1c_ goal^b^ (%)	Blood pressure (mmHg)
Healthy (few coexisting chronic illnesses^c^)	Longer remaining life expectancy	<7.5	<140/90
Complex/intermediate (3+ coexisting chronic illnesses^c^)	Intermediate remaining; life expectancy, high treatment burden, hypoglycemia, vulnerability, and fall risk	<8.0	<140/90
Very complex/poor health (long-term care or end-stage chronic illnesses^d^	Limited remaining life expectancy makes benefit uncertain	<8.5	<150/90

^a^ADA: American Diabetes Association.

^b^A lower A_1c_ goal may be set for an individual if achievable without recurrent or severe hypoglycemia or undue treatment burden.

^c^Coexisting chronic illness are conditions serious enough to require medications or lifestyle management and may include arthritis, cancer, congestive heart failure, depression, emphysema, falls, hypertension, incontinence, stage 3 or worse chronic kidney disease, myocardial infarction, and stroke.

^d^The presence of a single end-stage chronic illness, such as stages 3 and 4 congestive heart failure or oxygen-dependent lung disease, chronic kidney disease requiring dialysis, or uncontrolled metastatic cancer.

#### Aim 2: Overview

Compare patient weight loss and diabetes-related outcomes among those who receive obesity screening and counseling to those who do not, following implementation of preventive service coverage. We will examine postpolicy impact of obesity screening and counseling in a cohort of patients with diabetes and at increased risk for diabetes. Specific outcomes to be examined include weight loss, diabetes incidence, and diabetes outcomes (including HbA_1c_, controlled blood pressure, and use of a statin medication). Exploratory outcomes will include PROs. Furthermore, we will determine patient characteristics, including demographics (age, race/ethnicity, and rurality), and practice characteristics, including provider type, and their impact on receiving/providing obesity screening and counseling. Understanding patient and practice characteristics most likely to engage in obesity counseling can identify best practices and inform how to increase engagement by both patients and providers.

##### At Increased Risk Cohort Definition

The cohort of patients under study will be defined as patients aged 18 years and older who are at increased risk for the development of diabetes based on being overweight. Patients seen at one of the PaTH institutions will be included in the at increased risk cohort if they have a BMI ≥25 kg/m^2^, based on most recent recorded weight and at least one recorded height. The at increased risk cohort will be further limited to patients who will likely to be captured in the PaTH EHRs or claims data so that outcome assessments can occur. Thus, we will further limit the at increased risk cohort to patients who have either (1) had at least 2 outpatient primary care visits in one of the PaTH health systems in the past 3 years (since January 1, 2012) or (2) for whom claims data is available. The cohort will be dynamic, with new patients added into the cohort after 2015 as they meet the at increased risk cohort definition prospectively. Patients will not be removed from the cohort even if they are no longer overweight. The observational period for the outcome variables will be for the 10-year period from 2009 to 2019, thus including 3 years of data before the first policy change (CMS instituting coverage for IBT for obesity) under study (see Figure 2).

##### Definition and Measurement of Key Diabetes Outcomes and Covariates

The key diabetes prevention outcomes in this aim will be assessed on the population level. Specifically, we will examine (1) the impact of broader coverage for intensive behavioral counseling for obesity on counseling receipt in patients aged ≥65 years and (2) the impact of intensive behavioral counseling for obesity on counseling receipt in patients aged <65 years. Receipt of counseling for obesity will be assessed through PaTH EHRs and supplemented by claims data when available, utilizing G0447, G0473, S9470, and/or S9449 CPT codes in combination with a diagnosis of obesity (278.00, 278.01, 278.03, and 278.91, respectively; V85.3-V85.4). Key exposure variables will include individual level variables (sociodemographics and medical comorbidities). Medical comorbidities will be assessed using a modified Charlson Comorbidities Index adapted for use with the EHR.

### Stakeholder Engagement

**Figure 3:**
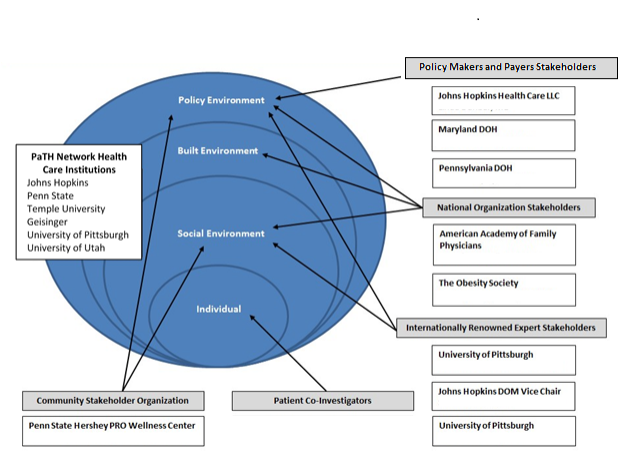
Conceptual framework and stakeholder advisory board members’ expertise. DOH: department of health; DOM: department of medicine.

We have created this research study with a focus on patient centeredness in all aspects of the research design. The following model (Figure 3) takes the conceptual framework and overlays the Stakeholder Advisory Board members’ expertise and their reach on both a regional and national level. As demonstrated, we have focused stakeholders on every layer of the model, ensuring successful engagement in all aspects necessary for the project. For example, we have 3 stakeholders with expertise and reach into the policy environment, including the Departments of Health from both Maryland and Pennsylvania. Representation from National Professional Organizations, including the American Academy of Family Physicians and The Obesity Society, offers expertise in policy, built, and social environments, in addition to networks for dissemination. Our engagement of internationally renowned diabetes researchers will offer important insight into shaping the research in all aspects and offering important assistance in dissemination. The tremendous reach of the Penn State Hershey PRO Wellness Center’s state-wide Advisory Board allows further expertise from patient-centered organizations and avenues for future dissemination. We will also include 6 PCPs who serve on the frontlines of this policy change.

Finally, we maintain patients at the very center, as evidenced by our patient coinvestigators, individuals experienced with diabetes and at increased risk for diabetes as both patients and caregivers. Our patient coinvestigators are ready to serve as the voices of patients afflicted with these diseases and feel equally committed to being involved in research:

It is vitally important for patients [to be involved in research]! Understanding more about diabetes and providing input to researchers about my personal experiences is mutually beneficial to the patient and the research.

Patient coinvestigator

### Statistical Analysis Plan

Descriptive statistics will be generated to describe the characteristics of different cohorts of interest. The diabetes-related outcomes will be summarized at the individual level on a yearly, quarterly, or monthly basis depending on the availability of the data within the data source. There will be binary outcomes (Yes/No) such as receiving obesity and/or nutritional counseling, controlled diabetes, controlled blood pressures, receiving annual eye exam, and count outcomes, such as numbers of clinic visits, emergency department visits, and hospitalization, and continuous outcomes, such as weight and HbA_1c_. The distributions of outcome measures will be examined by using minima, maxima, ranges, medians, quartiles, means, and SDs for continuous variables and frequency and contingency tables for categorical variables.

**Figure 4:**
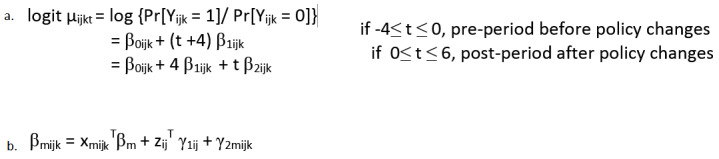
Statistical analysis equations.

To evaluate the impact of policy changes on these outcomes (Aim 1), we will examine how these outcomes change over time, in response to the policy changes. As descriptive analyses, we will plot the mean trajectory of each outcome over time at the clinic level, health system level, and state level. Outcomes will be at least annually, but possibly monthly or quarterly, depending on variable availability from the data source. The statistical modeling of patterns of changes in individual-level outcomes will be carried out through multilevel mixed-effects models [25-28]. The mixed-effects model is a common and popular modeling technique for longitudinal data. A mixed-effects model can accommodate within- and between-subjects variability, as well as serial correlations. In addition, it has the flexibility to incorporate time-dependent covariates, incomplete data, and heteroscedasticity of the variances and correlations. The mixed-effects model will be specified in a multilevel fashion so that different levels of variability (eg, individual characteristics and policy environment) can be taken into account. Specifically, state policies can be examined given the diversity of clinical locations and insurance expansion throughout the study timeframe. The pattern of changes in the outcome will be assessed for pre- and postperiods, respectively, based on the piecewise/segmented regression models. The slope of each segment indicates the trend of change in weight loss and diabetes outcome in that period. Therefore, the change in the trend/slope postpolicy implementation may reveal the actual impact of the new policy controlling for baseline level and trend. Such modeling strategies share the same spirit of interrupted time series analysis. Although the classical interrupted time series design often generates a single extended series of data, we have a large number of short series from each individual subject, namely, longitudinal data. Depending on the types of the outcomes, we will specify mixed-effects models based on logistic regression for a binary outcome, Poisson regression for a count outcome, and linear regression for a continuous outcome as detailed in equation a (Figure 4).

In the multilevel modeling, the first-level unit is the repeated measurement (at least annually, but potentially monthly or quarterly, dependent on variable availability) for the individual subject (pre- and postpolicy); the second-level unit is the individual subject; the third-level unit is the health system, provider, or clinic within the health system (cluster); and the fourth-level unit is the state. For example, let Y_i__j__k__t_ denote the binary response of receiving obesity and/or nutritional counseling at year t as broader coverage change in 2013 for the k^t^^h^ subject within the j^t^^h^ cluster of the i^t^^h^ state, t=–4, –3, ..., 0, 1, ..., 6, i=1, 2, 3; j=1, 2, ..., c_i_, and k=1, 2, ..., n_i__j_, where c_i_ is the number of clusters within the i^th^ state and n_i__j_ is the sample size within the j^t^^h^ cluster of the i^t^^h^ state. An individual subject may not be in the system for all the time points of measurement, so t will have a smaller range of values for that individual subject. The probability of receiving obesity and/or nutritional counseling, μ_i__j__k__t_ = E(Y_i__j__k__t_)= Pr[Y_i__jk_ = 1], can be described by equation b (Figure 4), a segmented logistic regression model where β_mi__jk_ (m=0, 1, 2) are subject-specific regression parameters, with β_0i__jk_ being the log odds of receiving obesity and/or nutritional counseling at t=−4, and β_1i__jk_ and β_2__i__jk_ being the slopes (annual change in log odds) for the pre- and postperiods, respectively.

In the framework of a mixed-effects model, each β_mi__jk_ is modeled by equation b (Figure 4) where x_mi__jk_ is the vector of regressors for the fixed effects, β_m_ is the corresponding vector of fixed-effects parameter coefficients, z_i__j_ is a vector of cluster-level regressors for the random effects for the j^t^^h^ cluster of the i^t^^h^ state, γ_1i__j_ is the cluster-level random-effect coefficients and is common to all m, and γ_2mi__jk_ is the subject-level random-effect coefficients associated with the parameter β_m_ for the k^t^^h^ subject within the j^t^^h^ cluster. The random effects are assumed to follow a multivariate normal distribution with mean zero. As the vector x_mi__jk_ may include subject-level, cluster-level, and state-level exposure variables, the fixed-effects parameter vectors β_1_ and β_2_ represent the effects of different exposure variables on the annual changes in the pre- and postperiods, respectively. Thus, we may perform statistical tests to examine whether there are differences in trends between the pre- and postperiods overall and for each state and whether the patterns of changes differ between the states.

Similarly, for a count outcome Y_ijkt_ (eg, number of clinical visits), we define the expected value μ_ijkt_=E(Y_ijkt_) and apply a Poisson regression model based on the natural log link function. The log expected number of clinical visits, log(μ_ijkt_) can be modeled with the aforementioned segmented mixed-effects model. The use of an offset term in the models yields the estimates of the rate of clinical visits rather than the mean number of visits. The fixed-effect parameters, β_1_ and β_2_, represent the effects of exposure variables on the annual changes in log of incidence rates for the pre- and postperiods, respectively. For a continuous outcome Y_ijkt_, we will model the mean, μ_ijkt_=E(Y_ijkt_), with the mixed-effects model, and the parameters β_1_ and β_2_ indicate the effects of exposure variables on the annual changes in the outcomes for the pre- and postperiods, respectively. Besides mixed-effects models, we will also consider marginal models based on generalized estimating equation approach. Although mixed-effects models in general yield subject-specific effects except for the continuous outcomes, marginal models yield population-level effects. All final statistical models will be assessed with regard to the goodness-of-fit and the appropriateness of model assumptions.

For Aim 2, we will compare patient weight loss and diabetes-related outcomes among those who receive obesity IBT to those who do not, following implementation of preventive service coverage. We will also investigate obesity counseling as a continuous variable to examine impact of intensity (defined as the number of sessions) on outcomes, including weight loss. The outcome data collected after the broader coverage change will be used for the analyses in Aim 2. To compare the trend of change in each outcome between 2 groups, we will use the mixed-effects models as described above. The indicator of receiving obesity IBT, time, and their interaction will be the primary variables of interest in the model. In addition, patient and practice characteristics will also be included as covariates to control for their effects on the outcomes. To incorporate different starting dates of IBT and multiple IBT over different years, we will use the time-varying indicator of receiving IBT in the models. For subjects who receive IBT, we will also examine how the intensity of IBT (number of visits) is associated with changes in the outcomes. The number of screening and counseling will be used as a predictor in the mixed-effects models. Patient and practice characteristics can be adjusted in the regression model to reduce the selection bias of receiving IBT. The propensity score matching method has also been widely used to balance the characteristics of those who receive IBT with those who do not. We will consider demographics, insurance coverage, access to IBT prescription, medical comorbidities, and information on use of health care services to calculate the propensity scores. In addition, we will consider health behaviors, which are available for analysis from some institutions, and include validated standardized questionnaires assessing nutritional intake (specifically fruit and vegetable consumption), physical activity, and sleep. An initial step within this project is to inventory the availability of PROs across institutions, and therefore, limited details are available at this time. A subject with IBT will be matched with a subject without IBT based on age, gender, enrollment window, and propensity of receiving IBT. Then mixed-effects models can be used to compare the patterns of changes in the outcomes of interest between 2 groups. Statistical software SAS 9.4 and R environment will be used to implement the proposed analyses.

#### Subgroup Analyses

Owing to the heterogeneity of the population and a dynamic cohort in our study, we will investigate subgroup analyses to assess how the policy impact varies across different subgroups. Following the general modeling approaches as described above, we will examine the benefits of policy changes for different subgroups including (1) patients with insurance throughout the study period, (2) patients who obtained insurance after the policy changes, (3) patients without insurance throughout the study period, (4) patients newly enrolled in the system after policy changes, and (5) other subgroups of interest according to gender, age (eg, aged ≥65 years), race-ethnicity, and rural status. Meta-analysis has been a powerful approach to combining the effects of interest across different studies, different populations, and different subgroups [29,30]. We will adopt this method to evaluate the average impact of policy changes across subgroups. A forest plot will be generated to reveal how the addition of a subgroup to the meta-analysis may affect the average policy impact.

#### Propensity Scores Matching

In the modeling framework above, we adjust for subject-level and cluster-level differences by including the exposure variables at different levels as covariates in the models. We will also consider a secondary analysis with a propensity score–matching approach to adjust for these differences [31,32]. A wide array of patient measures in the EHR, including demographics, insurance coverage, medical comorbidities, health behaviors, and information on use of health care services will be used to calculate the propensity scores. A propensity score–based stratification analysis will be performed to evaluate the overall impact of health policy using the modeling framework similar to that described above.

#### Analyses of Diabetes Outcomes at Population Level

The primary analysis of our study focuses on the individual-level outcomes. The statistical models yield the estimate of average change at the individual level post policy implementation. Given the information in the EHR data, we can also aggregate the diabetes outcomes at the community level and clinic level. For example, the proportion of patients with controlled diabetes can be obtained for each clinic and used as the outcome variable in the statistical modeling. The proposed mixed-effects modeling framework is still applicable in this case. The statistical analyses can be performed in a similar fashion to that for the individual-level outcomes.

For example, our statistical analysis plan can be easily modified to compare the differences in weight loss and diabetes outcomes (including diabetes control, controlled blood pressure, use of a statin medication, receipt of an annual eye exam and annual urinary microalbumin test, and lower extremity amputations) between rural and urban areas. Although our main analysis is on individual-level outcomes, aggregated outcomes at the community or county level can also be extracted from the EHR data. For example, the proportions of patients with controlled diabetes in each county at each year can be obtained and used as the outcome variable after arcsine-square root transformation in the statistical modeling. We can evaluate the rural/urban effects on the pattern of changes in diabetes outcomes over years by including county-level characteristics such as rural versus urban in the mixed-effects models as fixed effects. The time origin in the analysis will be the beginning of the study period rather than the time when insurance policy changes occurred. The counties sharing similar characteristics (eg, access to the same health system) will be considered as a cluster, and the clustering effect will be accounted for in the mixed-effects model analysis by including cluster-level random effects. Instead of using segmented regression models to evaluate the trend in diabetes outcomes before and after the policy change, we will consider linear or nonlinear trends in the diabetes outcomes and allow rural and urban counties to have different patterns of changes in the models.

Given that the study investigates a natural experiment, there is no primary data collection planned. However, there remains a risk of missing data. It is anticipated that the EHR will have incomplete data on outcomes of interest, which will be handled statistically as described below. Furthermore, the availability of claims data will assist in improving rates of missingness. Finally, we expect most missing data will be noninformative, that is, because of patients moving away from the health care institution. We will use validated statistical methods to handle missing data. These include the likelihood-based mixed-effects models to handle missing outcome data and multiple imputation method for missing covariates. The assumptions about missing data will be assessed based on the documented missing reasons and statistically as well. As participants are not recruited to this study, there will be no dropout to account for in the study design. However, given the prospective study design, there remains a risk for missing data. A consort-type diagram will be created to document each step to fully account for and justify patients who might be excluded. The missing data pattern will be summarized for primary outcomes. Baseline characteristics will be compared between those with missing outcome data and those without. A sensitivity analysis based on different assumptions of missing data mechanisms will be performed to evaluate the robustness of findings to the missing data.

#### Statistical Power

Owing to the very large sample sizes that are anticipated for the research studies (more than 320,000 patients with diabetes and 2,000,000 patients at increased risk for diabetes), there is tremendous statistical power to detect very small effect sizes for individual-level exposure variables. Therefore, the clinical investigators on this project will need to examine each statistically significant result and determine whether it is also clinically significant. Furthermore, such large sample sizes ensure the robust estimation results from the proposed multilevel statistical modeling, which involves a large number of regression coefficient and covariance parameters. The major benefit of the large sample size for each research study is that it provides sufficient statistical power for investigating effects of interest within subgroups that might be constructed according to age, race-ethnicity, and cohort decompositions.

## Results

Our PCORI-funded study is underway. To date, we have obtained our second data extraction from the PaTH CDRN and are performing data editing and cleaning. Next steps include analysis of early policy change (Multimedia Appendix 1).

## Discussion

### Overview of Proposed Findings

The overarching goal of this research is to understand the comparative effectiveness of obesity counseling as covered by CMS and other insurers in improving weight loss for adults either with or at increased risk of type 2 diabetes. As patients who are overweight are at highest risk for diabetes, improved weight management services could prevent diabetes and its negative health outcomes. Comparing weight and diabetes outcomes in 3 states using EHRs and claims data before and after this policy was implemented using the PaTH Network will allow important insight into policy effectiveness.

### Limitations of Research Design

#### Limitations of the Data

Through the PaTH network, we have access to EHRs for patients who have been seen at Penn State Hershey Medical Center, University of Pittsburgh Medical Center, Johns Hopkins University, and Temple University Health System and, beginning in Fall 2015, the University of Utah and Geisinger Health System. Although this a uniquely large integrated research network, it only includes patients who seek care at one of these large health systems or affiliates and does not include those who preferentially seek care outside of large health systems or do not seek care at all. We address this limitation, in part, by supplementing our health record data with claims data where available. We will enrich the PaTH data with the UPMC Health Plan, Temple Health Plan, Johns Hopkins Health System, Geisinger Health Plan, and University of Utah Healthcare claims data to ensure capture of outcomes data that occur outside of the PaTH health systems.

#### Data Integration

Our plans for data integration currently do not consider information extracted from images, videos, and free text, which can be important in some settings. However, our proposed design of the data infrastructure can be extended to incorporate such data in the future by leveraging state-of-the-art image analysis, video analysis, and natural language processing.

#### Residual Confounding

The possibilities of residual confounding cannot be ruled out because of unmeasured or unavailable factors. For example, no detailed data on individual behavioral risk factors (eg, diet and physical activity) are consistently available. Changes in individual behaviors will have large impacts on individual diabetes control. Similarly, data on built environment (eg, healthy food availability) are lacking. Finally, BMI and blood pressure are measured based on clinical practice not research protocol. They are subject to misclassification/measurement errors. Caution will, therefore, be exercised in interpreting our results.

## Appendix

Multimedia Appendix 1 Project milestones and timeline.

Multimedia Appendix 2 Peer-reviewed comments PCORI.

## Abbreviations

ADA: American Diabetes Association
BMI: body mass index
CDM: Common Data Model
CDRN: Clinical Data Research Network
CERC-DC: Comparative Effectiveness Research Core Data Center
CMS: Centers for Medicare and Medicaid Services
CPT: Common Procedural Treatment
EHR: electronic health record
HbA _1c_: hemoglobin A_1c_
IBT: intensive behavioral therapy
ICD: International Classification of Diseases
IRB: institutional review board
PCP: primary care physician
PCORI: Patient-Centered Outcomes Research Institute
PHI: protected health information
PNPRC: PaTH Network Protocol Review Committee
PRO: patient-reported outcome
SNOMED: Systematized Nomenclature of Medicine
UPMC: University of Pittsburgh Medical Center
