# Correction: A Patient-Centered PaTH to Address Diabetes: Protocol for a Study on the Impact of Obesity Counseling

**DOI:** 10.2196/17437

**Published:** 2020-03-04

**Authors:** Jennifer L Kraschnewski, Lan Kong, Erica Francis, Hsin-Chieh Yeh, Cindy Bryce, Jennifer Poger, Erik Lehman

**Affiliations:** 1 Penn State Health College of Medicine Penn State University Hershey, PA United States; 2 Johns Hopkins School of Medicine Johns Hopkins University Baltimore, MD United States; 3 University of Pittsburgh Department of Public Health Pittsburgh, PA United States

In the article “A Patient-Centered PaTH to Address Diabetes: Protocol for a Study on the Impact of Obesity Counseling” (JMIR Res Protoc 2019;8(4):e12054), there were three instances of incorrectly written code. The following corrections have been made:

In the Introduction section, under the subheading “Objectives”, under the bullet point of “Aim 1”, the code has been changed from “G0477” to “G0447”. The text was revised from:

We will determine how the annual probability of receiving obesity counseling (as defined by Common Procedural Treatment [CPT] codes G0477, G0473, S9470, and/or S9449)

to:

We will determine how the annual probability of receiving obesity counseling (as defined by Common Procedural Treatment [CPT] codes G0447, G0473, S9470, and/or S9449)

In the Methods section, under the subheading “PaTH Patients With Diabetes and At Increased Risk for Diabetes”, the code has been changed from “G0477” to “G0447”. The text was revised from:

For this study, receipt of IBT will include the presence of the G0477, G0473, S9470, and/or S9449 CPT codes

to:

For this study, receipt of IBT will include the presence of the G0447, G0473, S9470, and/or S9449 CPT codes

In the Methods section, under the subheading “Definition and Measurement of Key Diabetes Outcomes and Covariates”, the code has been changed from “G0477” to “G0447”. The text was revised from:

Receipt of counseling for obesity will be assessed through PaTH EHRs and supplemented by claims data when available, utilizing G0477, G0473, S9470, and/or S9449 CPT codes

to:

Receipt of counseling for obesity will be assessed through PaTH EHRs and supplemented by claims data when available, utilizing G0447, G0473, S9470, and/or S9449 CPT codes

These corrections do not impact the results or findings of the paper.

These corrections will appear in the online version of the paper on the JMIR Research Protocols website on March 4, 2020, together with the publication of this correction notice. Because this was made after submission to PubMed, PubMed Central, and other full-text repositories, the corrected article has also been resubmitted to those repositories.

