# Supplementary material for: A Patient-Centered PaTH to Address Diabetes: Protocol for a Study on the Impact of Obesity Counseling
Source: JMIR Res Protoc. 2019 Apr 4;8(4):e12054. doi: 10.2196/12054 (PMC6538312; doi:10.2196/12054)
Supplement: Multimedia Appendix 1 [file resprot_v8i4e12054_app1.pdf]

Table 8. Project milestones and timeline.

**Steps and Milestones**	**Year 1 (Q)**				**Year 2 (Q)**				**Year 3 (Q)**				**Year 4 (Q)**				**Year 5 (Q)**			
	1	2	3	4	1	2	3	4	1	2	3	4	1	2	3	4	1	2	3	4
Annual in-person meeting for Research Team	X				X				X				X				X			
Subcontract execution	X																			
Development and finalization of study protocol with key stakeholders	X	X																		
IRB Approval		X																		
Initial Data Extraction from PaTH to define cohort		X																		
Data editing of cohort data			X																	
Preparation of second data extraction request			X	X																
Interim Report to CDC				X																
Second Data Extraction from PaTH				X	X															
Data Cleaning and Editing					X	X														
Programming and determination of final data models						X	X													
Analysis of early policy change							X	X												
Manuscript preparation and submission of protocol							X	X	X	X										
Interim Report to CDC								X												
Manuscript preparation and submission of early analyses									X	X	X	X								
Interim Report to CDC												X								
Preparation of final data extraction request													X	X						
Final Data Extraction from PaTH															X	X				
Interim Report to CDC																X				

Data Cleaning and Editing																X	X			
Final analyses																		X	X	
Manuscript preparation of final results																		X	X	X
Dissemination materials developed																		X	X	X
Data reporting																				X
Final report																				X
