# Supplementary material for: A Patient-Centered PaTH to Address Diabetes: Protocol for a Study on the Impact of Obesity Counseling
Source: JMIR Res Protoc. 2019 Apr 4;8(4):e12054. doi: 10.2196/12054 (PMC6538312; doi:10.2196/12054)
Supplement: Multimedia Appendix 2 [file resprot_v8i4e12054_app2.pdf]

PATIENT-CENTERED OUTCOMES RESEARCH INSTITUTE

SUMMARY STATEMENT
(Privileged Communication)

**Principal Investigator:**	Jennifer Kraschnewski
**Organization:**	Pennsylvania State University Hershey Med Ctr
**Project Title:**	A Patient-Centered PaTH to Addressing Diabetes: Impact of State Health Policies on Diabetes Outcomes and Disparities
**PCORI Funding Announcement:**	Natural Experiments Network
**Review Cycle:**	PCORnet
**Request ID:**	NEN-1509-32304

In-Person Review Discussion Notes:

Strengths:

- Reviewers agreed that including Hemoglobin A1C (HbA1c) was a patient-centered outcome. Diabetic patients had responded that it was an outcome that mattered to them when asked in focus groups.
- Reviewers praised the weight loss and health services utilization outcomes as ones that are important to patients.
- The overall patient and stakeholder engagement plan was found to be a major strength of the application. Patients and other relevant stakeholders will be involved in all phases of the research. Their planned involvement in the dissemination and implementation was highlighted.

Weaknesses:

- Reviewers thought that additional factors that contribute to HbA1c should have been included to enhance the patient-centeredness of the research study. They felt that access to test strips and monitors as well as measures of patient education and activity levels were essential pieces of information that would enable better understanding of the impact of the health policy changes.
- Additional patient clinical outcomes, which may already be in the available data, such as lipid levels and cardiovascular events should have been included.
- Although the review panel considered HbA1c a patient-centered outcome, there was

suggestion by a few reviewers that improvements in HbA1c, rather than reaching the static target of HbA1c less the 7, should be considered as this is a more patient-centered goal indicating improvement in glycemic control. Additionally, the static target of HbA1c less than 7 may be unreachable for some patients.
- Reviewers suggested that the study sites should be expanded to include non-academic medical centers, such as community health centers, to ensure the study results are applicable to a broad range of patients.
- Despite the overall strength of the engagement plan, reviewers expressed several minor concerns that are easily addressable. They were unclear regarding the number of patients on the advisory board and wanted further clarification. There was some concern that there should be more than four patient co-investigators to be truly representative of the range of patients who will be seen and to make sure there is adequate input throughout the life of the study. Reviewers also wanted further details of how patient and stakeholder input will be incorporated into the planned study design.

Additional Comments:

- Reviewers also noted that the states involved in the study may each change their Medicaid coverage during the course of the study. They suggested applicants consider how these changes could impact the study plans and possibly be capitalized upon to generate additional useful information.

The following reviewer critiques were completed prior to the in-person review and were not altered post-discussion.

Criterion 1: Patient-centeredness

Reviewer 1:

Strengths:

- The proposed work to determine if an improvement in outcomes results from policy changes through the ACA and CMS is well justified and addresses an issue important to diabetics as they address the utilization of services of which these patients are always concerned.
- It addresses one of the key patient-centeredness criteria of PCORI which is “how can clinicians and care delivery systems they work in help me make the best decisions about my health and health care”? This will be done by studying the impact of the ACA and CMS objective for Diabetes 2020 objectives for diabetes and diabetes prevention which is likely going to have a far reaching public health impact. This is a major strength of the study.
- The study makes a good case for the fact that significant health disparities exist in the care of diabetics with racial and ethnic minorities having a higher rate of complications and mortality. This is in line with the PCORI question of “Given my personal characteristics, conditions, and preferences, what should I expect will happen to me?” This study will help answer the question if truly population-based interventions help improve diabetes outcomes in these subsets of patients.
- Aim 1. Hemoglobin A1c remains an important patient-centered outcome, given it is well-established that improved glycemic control results in prevention of serious complications (e.g., cardiovascular disease, blindness, renal failure). In addition the proposal will examine blood pressure control, use of a statin medication, and appropriate diabetic screening, given the

importance of these guideline-recommended measures in diabetes care. The PI mentions that it is established that these outcomes are of interest to patients. This is a moderate strength.

Weaknesses:

- The study leverages the PCORI funded PaTH CDRN network whose members are four academic institutions across three states. This leaves out a large number of states that may not have such academic programs and the validity of the study across nonacademic centers comes into question. Also the characteristics and population of patients in the other areas might be significantly different from those getting care at the academic centers, thus raising the question if the study results can truly be considered "population based". This is a minor weakness. It can be remedied by including more states and community health centers in future studies.
- There is no mention of preliminary data from patient focus groups to verify if this is a topic of interest to patients and caregivers .There is no preliminary data either to suggest health policy based interventions are of interest to caregivers or insurers either who would be major stakeholders interested in this study outcomes. This is a minor weakness.

Reviewer 2:

Strengths:

- The research team conducted focus groups with patients who are obese and have diabetes to identify research questions of interest. Thirteen participants revealed they were interested in having counseling provided in a primary care setting. The data collected in this formative work allowed the researchers to design a patient-centered study that focused on research patients care about.
- Patient stakeholders were instrumental in aligning the needs of patients in this study with the Healthy People 2020 Goals as they refined the proposal for the PCORI submission.
- The proposal addresses all four of the key questions of the PCOR definition. Each component (patient preference, healthcare choices, important outcomes, and decision making) is described in detail.
- The patient partners informed several measures in the study. For example, patients wanted A1c to be a measure and thought it was "patient-centered".

- The patients on the team were instrumental in the development of Aim 2 in the proposal. These patients wanted to add weight loss as a measure and described it as being an important outcome.

- A project timeline demonstrates how the study is patient-centered as well as engaging over the course of the project; each milestone described indicates how and when patients will be involved. It shows the study is centered on the patient throughout all 5 years of the project.

- The proposal describes how the research will meet the PCORI definition for patient-centeredness. The team describes how it will evaluate a topic of interest to patients and will help patients learn about how insurance can impact their diabetes care (both benefits and harms).Furthermore, the study will focus on helping patients make decisions regarding their care and insurance. Additionally, the proposal describes how the study will measure weight loss and A1C, which patients state are important.

Weaknesses:

- It is unclear why the research team states it cannot use a patient reported outcome in the study. In the CDC application it states PCOR PATH CDRN uses a questionnaire as a component of the system to capture PROs - that system is being used in this study.

<u>Reviewer 3</u>:

Strengths:

- The proposal tests one of the most fundamental questions of patient-centeredness of our time – does Medicaid expansion impact access to care, care decisions, the care itself, and ultimately the lives of individuals, in this case, individuals with pre-diabetes and diabetes.
- Similarly, the proposal tests whether coverage of preventive care, mandated by the ACA, impacts weight loss and obesity counseling resulting in individual patient's weight loss.
- The proposal, in theory, tests all of the elements of PCOR. Individual's personal characteristics, conditions and preferences should expect broader access to and options for care under Medicaid expansion when compared to those who do not have broader access, or whose access is delayed.
- Medicaid expansion offers better access to preventive, primary, and chronic disease care, the potential benefits of which should be prevention and earlier treatment of illnesses. Although there are potential harms from medical care, presumably the benefits should outweigh the harms of those options, leading to improved outcomes of those receiving earlier and broader care.
- Of all of the PCOR outcomes, access to a broader array of care choices under Medicaid will allow clinicians and the care delivery systems they work in help individuals make the best decisions about their health and healthcare. No healthcare coverage at all, or coverage under high deductible/cost share plans, severely limits individual's care options, often offering them no options at all. The leading cause of personal bankruptcies in the U.S. today is cancer and the personal costs of treating it. Patients in these circumstances are faced with the choice of a life saving treatment or food and shelter for their families. Individuals covered by Medicaid rarely, if ever, face such stark choices. This study offers the option of investigating the burden placed upon the citizens of one state choosing not to expand Medicaid as compared to a state choosing to expand late and compared to an early expansion state.

Weaknesses:

- The strengths above are strengths in theory; it is not clear if they intend to look at personal health information to be able to isolate and measure these questions of patient centeredness. The study compares HbA1c results in citizens in early, late and non- Medicaid expansion states. This is very important information, but is not patient centered in a way that looks at what individuals can expect from their care, their care options, both good and bad, outcomes of care and how the healthcare system adapts to improve care. HbA1c is an excellent marker of diabetes care outcomes over time, but it does not explain how those outcomes came about.
- The researchers seem to have access to data on weight loss, suggesting that they have access to more than just claims data. If so, the researchers ought to be able to access other individual, non-claims based data besides HbA1c. Such information would offer information on the impact of many factors that lead to improvements or deterioration of HbA1c. For example, does Medicaid expansion allow individual's, previously covered by high deductible insurance

plans, to better afford blood sugar test strips or refills of insulin or other medications? If access to such data is possible, the researchers seem not to intend to use this information. The study has numerous and considerable strengths, but this is a significant weakness.

Reviewer 4:

Strengths:

- Type II diabetes can be postponed or prevented with life changes, such as obesity, nutrition, activity, and diabetes education. This study looks at obesity, which is a moderate strength. However, the study would be more significant if it encompassed broader patient change training.
- A moderate strength of this proposal is that the project has objectives that are clear and relevant to public health. The proposal seeks to identify the impact of health insurance extensions and obesity counseling. This is of interest to payers and health officials at a macro level, state by state, opting into Medicare expansion and obesity counseling.
- Patient interest is personal. Patients experience heath care at the one-on-one level with a caregiver. The investigators should provide more clarity on how the proposal will result in individual patient options for choice. This would be valuable as patients are not likely able to choose the inclusion of available obesity counseling or Medicare expansion at the state level.
- It is a moderate strength that the proposal seeks “more intensely” engaged patient partners. The proposal states it has had success in doing this. However, it is not exactly clear how patient and stakeholder inclusion has helped shape the research objective; this information would be valuable. The proposal notes that the patients participate in the process weekly and biweekly, but it is unclear what the contributions of those patients have been, what the expected contributions are, and what specific patient driven outcomes are being investigated.
- There is a link between obesity and diabetes, and that is a moderate strength. A broader definition of patient education that includes diabetes education would strengthen the proposal to a major strength. Diabetes self-care education is an underutilized Medicare benefit and seems highly relevant to this line of investigation. The project would be stronger with a clear definition of how individual patients choose to participate with counseling and education, and the value of that choice at the individual level.

Weaknesses:

- The project considers various responses to health care change, based on the regional choices made of policy makers in multiple states. Knowing differences in outcomes is valuable from a public health perspective, but may not facilitate individual patient health choice beyond relocation. It is unclear exactly how this project falls into the definition of PCOR. The project would be stronger with a clear definition of how it would help people and their caregivers communicate and make informed health decisions, allowing their voices to be heard in assessing the value of healthcare options. From a patient perspective this is a significant shortcoming, we the patients have little ability to change regional responses other than the political process.
- One key diabetes outcome stated is A1C < 7. That is a static measure (set not by this study but by Healthy People 2020). Diabetes is a progressive condition as changes to healthier lifestyles are also progressive. Success in modifying behaviors may significantly enhance A1C outcomes, but still fall above the target. Capturing individual patient progress and success towards their individual goals may be as valuable as a static goal.
- Defining success with type II diabetes should reflect patient progress. Under the proposed measure successfully making changes to reduce A1C from 10 to 8 would be seen as patient failing to succeed when, in fact, a very significant successful outcome was achieved. For

example A1C <7 or a semi-annual reduction in A1C of 10% or .7 whichever is greater. As a patient, I see this static goal as a real weakness. It is set outside of this study, which lessens the impact. This is a minor weakness.

- The proposal states an appreciation of diversity, but 25% non-white seems to understate the representation of people of color. Underrepresentation of non-whites is a significant weakness. Better diversity would strengthen the proposal, particularly as diabetes disproportionally impacts some communities of color. Rates of diabetes are higher in minority populations, therefore, to be patent-centric the project should better reflect the patient population.
- Care guidelines in diabetes emphasize the importance of individualized care programs. This study seems to have a weakened patient-centricity in which facilitating individual care plans is not part of the larger public health processes being investigated here. This is a moderate shortcoming.

Criterion 2: Patient and stakeholder engagement

<u>Reviewer 1</u>:

Strengths:

- One of the major questions to answer in the PCORI criteria to meet patient and stakeholder engagement is "Does the study include a description of how the CDRN is working with its health systems leaders or health plan collaborators around diabetes care?" The proposal leverages the novel infrastructure of the PaTH Clinical Data Research Network (CDRN) across three states who have instituted Electronic Health Records (EHR) based data infrastructure to share and study the health policies being studied in this proposal. This is a major strength.
- In specific preparation for the proposed study the investigators assembled a Stakeholder Advisory Board consisting of patients, clinicians, state agencies, and national patient advocacy and professional organizations. They engaged these Stakeholders in the development of this proposal and their input has shaped the study design. Stakeholders will continue to meet with the research team throughout the project duration to oversee study conduct and they report looking forward to better understanding recent policy change on diabetes outcomes.
- There is a clear plan with regards to patient and stakeholder engagement. Investigators will conduct quarterly one on-one meetings between Patient Co-Investigators and study staff to provide regular monitoring to ensure they feel meaningfully involved in the study and that they are equal contributors to the team. In addition, they will ask Patient Co-Investigators to facilitate these meetings with Stakeholder Advisory Board members, allowing for open responses from stakeholders as opposed to having Faculty Investigators responsible for these meetings. They will also utilize technology, an online collaborative platform, when appropriate to reduce stakeholder burden for participation. They will use a secure Stakeholder Advisory Board portal to collect and provide study information.

Weaknesses:

- There are only four patient co-investigators for the life of the study across the four academic centers. This number seems too small to get meaningful input and can be remedied by including more patient co-investigators. This is a minor weakness
- There is no clear guidelines in the proposal as to how the patient and stakeholder feedback will be incorporated in the ongoing study re design and restructure if needed based on the feedback. This is a minor weakness and can be remedied easily by giving some clarity on the

roles of the stakeholders.

Reviewer 2:

Strengths:

- The proposal describes how patient stakeholders have been involved in the formulation of the research question and in the development of the project aims. Additionally, the stakeholders have been involved in the selection of important outcomes such as A1c and weight loss.

- The role of each group of individuals on the team is detailed in the proposal. Patient Co-investigators, the Advisory Board and the Investigative team, have distinct roles and these roles seem to be of equal importance.

- There are four patient Co-investigators who will be more engaged and involved than typical patient partners in the project. These individuals will attend routine meetings, shape the research as it is conducted and will have decision making power along with the other members of the team.

- Nine stakeholders will serve on an advisory board, comprised of representatives from national organizations as well as patients. This group will oversee the project from development to dissemination.
- The proposal describes how all different types of stakeholders (patients, clinicians, national advocacy groups, professional organizations, payers, and policy makers) are engaged.

- The proposal describes how patient co-investigators will be educated on the research process and will be regarded as valued team members. The proposal describes how the Patient Co-investigators will be given a co-learner or “buddy” to work with as they work on the investigative team. Patient Co-investigators will be regarded as partners in the endeavor and will be paid just like the professional Co-Investigators. As the team is being trained, all team members will be asked to conduct themselves so that they trust one another, are transparent and honest.

- The proposal describes how the research team will work with Health System Leaders to deliver high value care by meaningfully engaging participants in the PaTH Network. This group will assist with dissemination of the findings.

Weaknesses:

- None.

Reviewer 3:

Strengths:

- The proposal discusses patient and health system stakeholder input into all aspects of the design, implementation, monitoring, and analysis and dissemination of results.
- The study proposal states that patients and other stakeholders are partners in formulating research questions, defining central aspects of the study design, choice of study participants, outcomes of importance to individuals, and overall conduct of the study. Key stakeholders are identified who will assist in dissemination and implementation activities; the roles and the

decision-making authority of all research partners are very clearly stated and the proposal does demonstrate the principles of reciprocal and partnering relationships, and co-learning.

- The study specifically describes how the CDRN is working with its health systems leaders and health plan collaborators around diabetes care.

Weaknesses:

- The CDC proposal does not mention stakeholder engagement in any detail, suggesting that patient-centeredness and stakeholder input were afterthoughts in the research design.
- Although the researchers say that stakeholder input informed the research design at all stages, examples of this are largely missing other than personal statements about the importance of a patient's weight loss to them, or how important knowing their HbA1c is to an individual with diabetes.
- Although the relationships of the study partners, how the principles of reciprocal relationships will be used are clearly described, examples of these aspects are lacking, which I consider a moderate weakness.

<u>Reviewer 4</u>:

Strengths:

- The project states plans for significant patient and stakeholder involvement. However, it is unclear what stakeholder and patient outcomes are being investigated. Including patient and stakeholder participation is a moderate strength of this study. However, including more clarity on what the patients have contributed toward the investigation outcomes would add to that strength. Adding the stakeholders' views on reaching outcomes, specifically including educators' views on patient programs, would also add value.
- The PaTH CDRN is proposed as part of the research effort. The proposal would be stronger if it identified how the CDRN and health systems are partnering with the research investigators in that process. Aim three seeks to identify diabetes services; it specifically mentions the ED and hospitalization. However, it would be stronger if the study aim also included and measured the use of patient-centered services such as obesity, nutrition, lifestyle, and education interventions that can slow the onset of type II diabetes. If this were added, it could make this a more significant strength.

Weaknesses:

- It is not abundantly clear how patients are engaged in defining relevant outcomes and how those outcomes being considered are patient-centered. Patient engagement is significant, therefore having clarity on patient defined research questions, outcomes, and other study parameters would strengthen this proposal.

Overall Comments

<u>Reviewer 1</u>:

This application proposes to understand the impact of expanded health insurance coverage and

preventive services through the Patient Protection and Affordable Care Act (ACA) and the Centers for Medicare and Medicaid (CMS) on Healthy People 2020 objective of diabetes and diabetes prevention. The proposal has the potential for high impact on public health. The proposal is patient-centered in development of its three Aims including the key outcome of reduction in A1C which they have shown is of concern to patients as well as weight loss and health utilization related to diabetes. This is a major strength. The study makes a good case for the fact that significant health disparities exist in the care of diabetics with racial and ethnic minorities having a higher rate of complications and mortality. The study has a strong, well-defined plan for patient and stakeholder engagement with regular participation and engagement. This group is also meaningfully engaged in the dissemination for the study results.

The study has a few minor weaknesses which can be readily remedied. The fact that it is utilizing EHR data from four large academic centers across three states raises concern about validity and applicability of these results to smaller communities served by non-academic institutions that may or may not have similar facilities. Inclusion of a community health center or non-academic institution could be considered by study investigators. Another minor weakness is lack of mention of pre-study data if outcomes related to health policy improvement and population health based interventions are of interest to patients and caregivers. Some preliminary data to demonstrate this would make the study more aligned with PCORI goals. Another minor and easily corrected weakness is the inclusion of only four patient co-investigators in a study of this large magnitude thus bringing to question if they would be able to represent all patient viewpoints. There also needs to be some more clarity on how the patient and stakeholder feedback will be incorporated in to the ongoing study redesign and how the investigators will address any changes they need to make based on the feedback. The investigators can provide more clarity on this by simply elaborating more on this in the proposal.

<u>Reviewer 2</u>:

Overall, the proposal describes how patients and other stakeholders will play significant roles in the project. The project is patient centered – it is designed around the desires and needs of the patient. Most notably, the project timeline is described in terms of the patient's role.

Moreover, the type and nature of the engagement described is logical and appropriate for the project. All members of the team seem to be genuinely valued and fully engaged in the proposal. However, the proposal does not justify why the project cannot include a patient reported outcome. This is a negligible weakness that could be easily corrected if the research team feels the change is appropriate. Ultimately, this is an outstanding proposal that when funded will provide valuable information to understand how the ACA and CMS changes impact diabetes care.

<u>Reviewer 3</u>:

This is a natural experiment using three states; one is an early Medicaid expansion state under the ACA, another is a late expansion state, and the third is a state which will not expand its Medicaid program. The research partners are well situated to test the impact of Medicaid expansion, a question of enormous political, social, and healthcare impact. To say that this question will be one of the central points upon which the next presidential election will be determined is not hyperbole. Their second question, the impact of obesity counseling, while not as politically prominent, is of major import as well. Further, the researchers have a network that allows these questions to be viewed through a patient-centered lens.

The researchers seek to test the impact of Medicaid expansion on diabetes care and personal outcomes of individuals with diabetes using measurement of HbA1c as the measure of personal

outcome. HbA1c is a long-term measure of glycemic control, which reflects the ultimate impact of a whole host of short and intermediate term personal social, medical, nutritional and other factors. Researchers looking at only medical claims on a population level use HbA1c to look at outcomes across a population. This research group has access to data and records of people in 3 states that promises to help explain which of these many factors seems to most positively and negatively impact glycemic control in the context of Medicaid expansion, as well as weight loss in the context of expansion of preventive care under the ACA. Further, the researchers have insight into conditions that affect those with pre-diabetes who go on develop diabetes.

The research team includes a varied group of stakeholders who will help design the research protocol. The proposal lacks clear examples of how these stakeholders will be used but promises to make use of their insight. This is potentially a powerful study. Many factors impact glycemic control; the stakeholder group appears to have the insight to be able to compare the effects of these many factors. The proposal asserts that stakeholder input was used in each stage of the proposal design, however examples of how the team was used would strengthen the proposal immeasurably. The only example offered seems to be the choice of HbA1c as a measure; examples of the stakeholder's ideas on measures that impact glycemic control would have been informative. The proposal states that the role of stakeholder input was added to every aspect of the original proposal which somewhat calls into question the researchers commitment to patient and stakeholder input.

Reviewer 4:

This appears to be a useful and valuable population health study. It is much less clearly a study that falls into PCOR. Ultimately, the outcomes will be significant for choices made by governors and state legislatures that is well beyond the scope of individual patients and their care teams.

This project is a useful evaluation of large-scale public health choices made by state policymakers. That certainly is useful in the context of initiatives by CDC and CMS. CDRN's can support this kind of valuable public health initiative. It is not clear that the CDRN and health systems have significant roles in collaboration with the project, and more detail would be useful.

The direct line to relevance to individual patients is even less clear than the value to policy makers. The choices the study evaluates are not patient-centered decisions; they are policy choices. From a patient perspective, the measurements of satisfaction with obesity counseling and patient-defined measures of success of such program would be valuable study considerations.
